# Trends in Opioid Misuse among Marijuana Users and Non-Users in the U.S. from 2007–2017

**DOI:** 10.3390/ijerph16224585

**Published:** 2019-11-19

**Authors:** Sunday Azagba, Lingpeng Shan, Lauren Manzione, Fares Qeadan, Mark Wolfson

**Affiliations:** 1 Department of Family and Preventive Medicine, University of Utah School of Medicine, Salt Lake City, UT 84108, USA; lingpeng.shan@utah.edu (L.S.); lauren.manzione@utah.edu (L.M.); fares.qeadan@utah.edu (F.Q.); 2 Department of Social Medicine, Population and Public Health, University of California, Riverside School of Medicine, Riverside, CA 92501, USA; Mark.Wolfson@medsch.ucr.edu

**Keywords:** prescription opioid misuse, marijuana use, prescription drugs, substance abuse

## Abstract

Prescription-opioid misus e continues to be a significant health concern in the United States. The relationship between marijuana use and prescription-opioid misuse is not clear from the extant literature. This study examined national trends in prescription-opioid misuse among marijuana users and non-users using the 2007–2017 National Survey on Drug Use and Health. Cochran–Armitage tests were used to assess the statistical significance of changes in the yearly prevalence of prescription-opioid misuse and marijuana use. Multivariable logistic regression was used to examine the association between prescription-opioid and marijuana use adjusting for sociodemographic characteristics. From 2007 to 2017, marijuana use increased, while prescription-opioid misuse declined. Larger declines in prescription-opioid misuse were found among marijuana users than non-users. Marijuana ever-use was significantly associated with prescription-opioid misuse. Specifically, marijuana ever-users had higher odds of prescription-opioid misuse (ever-misuse [OR: 3.04; 95% CI, 2.68–3.43]; past-year misuse [OR: 3.44; 95% CI, 3.00–3.94]; and past-month misuse [OR: 4.50; 95% CI, 3.35–6.05]) compared to marijuana never-users. Similar results were found for the association of past-year and past-month marijuana use with prescription-opioid misuse. This study provides data on trends and associations about opioid misuse among marijuana users and non-users in a changing social environment of drug use in the United States. Future research should consider whether there is a causal relationship between marijuana use and prescription opioid misuse.

## 1. Introduction

The opioid crisis was declared a public health emergency in 2017 by the U.S. Department of Health and Human Services [1]. This declaration came in response to the estimated 130 opioid overdose deaths occurring each day in the United States [2]. In 2018, about 10.3 million (3.7%) people had misused opioids in the past year, which was a small but statistically significant decrease from 4.2% in 2017 [3]. In 2018, 92.1% of opioid misusers (9.4 million) were pain reliever misusers [3]. The total economic burden of prescription-opioid misuse is estimated to be $78.5 billion per year in the United States [2]. Among patients prescribed opioids, those with a mental health diagnosis, younger age, male sex, and previous substance use are most likely to develop habits of misuse [4].

As the nation contends with the opioid crisis, U.S. marijuana use is on the rise. Marijuana is the most widely used illicit drug in the United States, and fewer adults perceive marijuana use as risky today than in the early 2000s [5,6]. Parallel to the changing public perception, there has been an increase in the number of states that have legalized marijuana [7]. U.S. marijuana use has also increased, with 43.5 million people (15.9%) aged 12 and older reported to have used marijuana in 2018 [3,6]. Frequent marijuana use has been associated with physical and mental health consequences, such as impaired breathing, increased heart rate, anxiety, depression, and psychotic disorders [8,9,10].

Many previous studies have examined the associations between marijuana and opioid misuse, with mixed results. For instance, Bachhuber et al. (2014) found that state medical cannabis laws were associated with lower state-level opioid overdose mortality rates from 1999 to 2010 [11]. However, a recent study was unable to replicate such results using more extensive data [12]. Other studies have also found little evidence of an association between medical marijuana laws and prescription-opioid misuse or related disorders [13]. There is some evidence, though, that those who used medical marijuana used fewer prescription drugs, including opioids [14,15]. Some studies found that marijuana use was associated with increased rates of successful opioid-dependence treatment [16,17]. However, Campbell et al. (2018) found no evidence that marijuana use improved patient outcomes and Caputi et al. (2018) found that medical marijuana users had a higher likelihood of reporting non-medical use of prescription pain relievers [18,19]. Prior studies have also shown that marijuana use was associated with increased odds of other drug use, including opiates [5,20,21]. However, another study found that the majority of people who use marijuana do not go on to use other drugs [22]. Little is known about the trends of prescription-opioid misuse among marijuana users and non-users. In the present study, we examined national trends in prescription-opioid misuse among marijuana users and non-users, and associations between prescription-opioid misuse and marijuana use.

## 2. Materials and Methods

### 2.1. Data

Data were drawn from the 2007–2017 National Survey on Drug Use and Health (NSDUH) (*N* = 617,121). NSDUH is a nationally representative, annual survey of the U.S. population aged 12 years and older. NSDUH, which is supported by the Substance Abuse and Mental Health Services Administration (SAMHSA), collects information from residents of households, non-institutionalized groups, and civilians on military bases [23]. Questions include age at first use, as well as lifetime, annual, and past-month use of the following drugs: alcohol, marijuana, cocaine (including crack), hallucinogens, heroin, inhalants, tobacco, pain relievers, tranquilizers, stimulants, and sedatives. The survey covers substance abuse treatment history and perceived need for treatment, utilizing questions from the fourth edition of the Diagnostic and Statistical Manual of Mental Disorders (DSM), which allows for diagnostic criteria to be applied [24]. Respondents are also asked about personal and family income sources and amounts, health care access and coverage, illegal activities and arrest records, problems resulting from the use of drugs, perceptions of risks, and needle sharing. Demographic data that are collected include gender, race, age, ethnicity, education level, job status, income level, veteran status, household composition, and population density. The weighted response rate ranged between 67.1% and 75.6% from 2007 to 2017. The trend analysis sample included 617,121 participants from 2007 to 2017. In the multivariable analysis, the sample was restricted to the 2016/2017 data (*N* = 85,179).

### 2.2. Measures

Dependent variable: prescription-opioid misuse was the main outcome of interest. Three dichotomized variables (ever-use, past-year use, and past-month use) were created to measure prescription-opioid misuse. The survey participants were asked: “Have you ever, even once, used any prescription pain reliever in any way a doctor did not direct you to use it?”; “In the past 30 days, that is, from <date> up to and including today, did you use <specific pain relievers> in any way a doctor did not direct you to use <specific pain relievers>?”; and “In the past 12 months, did you use <specific pain relievers> in any way a doctor did not direct you to use it?” However, participants were only asked for overall pain reliever misuse before 2015.

Independent variables: the main independent variable was marijuana use status. Three dichotomous variables (ever-use, past-year use, and past-month use) were created to measure marijuana use based on the survey question “How long has it been since you last used marijuana or hashish?”

Other substance use measures (i.e., cigarettes, alcohol, cocaine, crack, heroin, and methamphetamine) were assessed using the corresponding questions on ever-use status. Psychological distress was measured using the Kessler Inventory [25]. Participants aged 18 years of age and older were asked six questions on the frequency of feeling “nervous,” “hopeless, restless or fidgety,” “sad or depressed that nothing could cheer you up,” “everything is an effort,” and “down on yourself, no good, or worthless.” For each of the six questions, responses were coded on a scale from 4 (all of the time) to 0 (none of the time). A dichotomized psychological distress indicator (Yes: ≥13; No: <13) was created based on the sum of the six items. In addition, demographic characteristics were obtained from the data, including age (12–17, 18–25, 26–34, 35–49, and 50+), sex (male and female), marital status (married, widowed, divorced or separated, or never married), race (non-Hispanic White, non-Hispanic Black, Hispanic, and other races), education (less than high school, high school graduate, some college/associate degree, and college graduate), employment status (employed full-time, employed part-time, unemployed, and other), total family income (less than $20,000, $20,000–$49,999, $50,000–$74,999, and $75,000 and more), and overall health (excellent, very good, good, and fair/poor).

### 2.3. Statistical Analysis

We generated estimates of the prevalence of ever, past-year, and past-month prescription-opioid misuse and marijuana use for each survey year. Additionally, estimates of ever, past-year, and past-month prescription-opioid misuse were calculated for subgroups stratified by marijuana ever, past-year, and past-month use status. Chi-square tests were used to test the significance of prevalence differences between subgroups stratified by marijuana use status (past-month, past-year, and ever) for each year. In the trend analyses, Cochran–Armitage tests were used to assess the statistical significance of changes in the yearly prevalence of prescription-opioid misuse and marijuana use over time.

For 2016–2017 survey participants aged 18 and older, demographic characteristics and substance use were reported with unweighted counts and weighted percentages and described by the marijuana ever-use status (marijuana never-user, marijuana ever-user). Rao–Scott Chi-Square tests were used to compare characteristics between the two groups: marijuana never-users versus marijuana ever-users. Multivariable logistic regression was used to examine the association between prescription-opioid and marijuana use adjusting for age, sex, race, overall health, education, employment status, family income, survey year, past year severe psychological distress, and substance use, including cigarettes and alcohol. Sampling weights and survey strata were included in all analyses to account for the complex survey design. All tests were two-sided, and a *p*-value <0.05 was considered significant. We performed all data analyses using SAS version 9.4 (SAS Institute, Inc., Cary, NC).

## 3. Results

Descriptive characteristics of the 2016–2017 U.S. non-institutional population aged 18 and over are presented in Table 1. Among the 85,179 respondents from the 2016–2017 survey, 41,628 (52.5%) never used marijuana, and the remaining 43,551 (47.5%) ever used marijuana. The majority of participants were married, non-Hispanic Whites, high school graduates, and employed full time. Regarding substance use, 57.3% reported smoking a cigarette, 80.6% ever had an alcoholic beverage, 14.7% ever used cocaine, and 23.3% ever used crack. There are several differences between marijuana never-users and marijuana ever-users. Among marijuana never-users, 43.4% were at least 50 years old, 55.1% were female, 27.4% were never married, and 20.3% were Hispanic. About 38.3% of marijuana ever-users were at least 50 years old, 53.0% were male, and 35.0% were never married. In addition, substance use (i.e., cigarettes, alcohol, crack, heroin, and methamphetamine) was prevalent among marijuana ever-users (Table 1).

**Figure 1:**
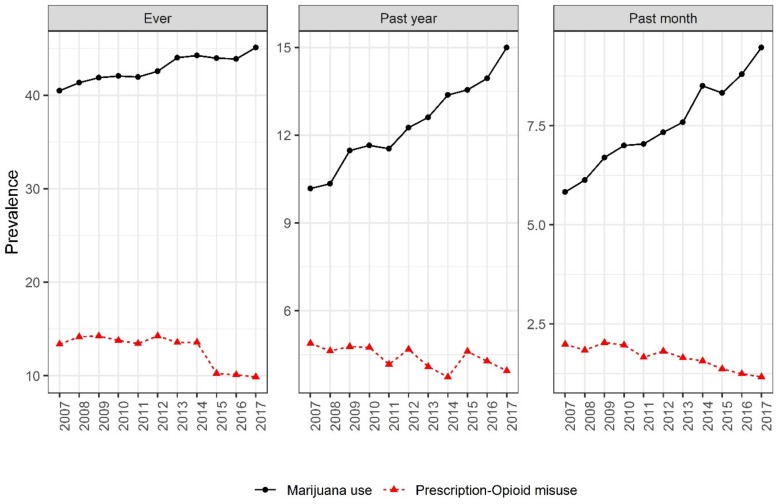
Prevalence of marijuana use and prescription-opioid misuse in the United States, 2007–2017. Cochran–Armitage tests were used to assess the statistical significance of changes over time (*p* < 0.001).

Figure 1 presents trends of marijuana and prescription-opioid use from 2007 to 2017. Across the study period, the prevalence of marijuana use increased significantly. Ever marijuana use (40.5% to 45.1%), past-year marijuana use (10.2% to 15.0%), and past-month marijuana use (5.8% to 9.5%) all increased from 2007 to 2017 (z = 1424.8, *p* < 0.0001; z = 2298.8, *p* < 0.0001; and z = 2174.6, *p* < 0.0001, respectively). In contrast, prescription-opioid misuse displayed the reverse trend from 2007 to 2017. Prescription misuse declined between 2007 (13.4%) and 2017 (9.9%) for ever misuse, past-year misuse (4.8% to 3.9%), and past-month misuse (2.0% to 1.2%) (z = 1127.1, *p* < 0.0001; z = 649.2, *p* < 0.0001; and z = 2152.5, *p* < 0.0001, respectively).

**Figure 2:**
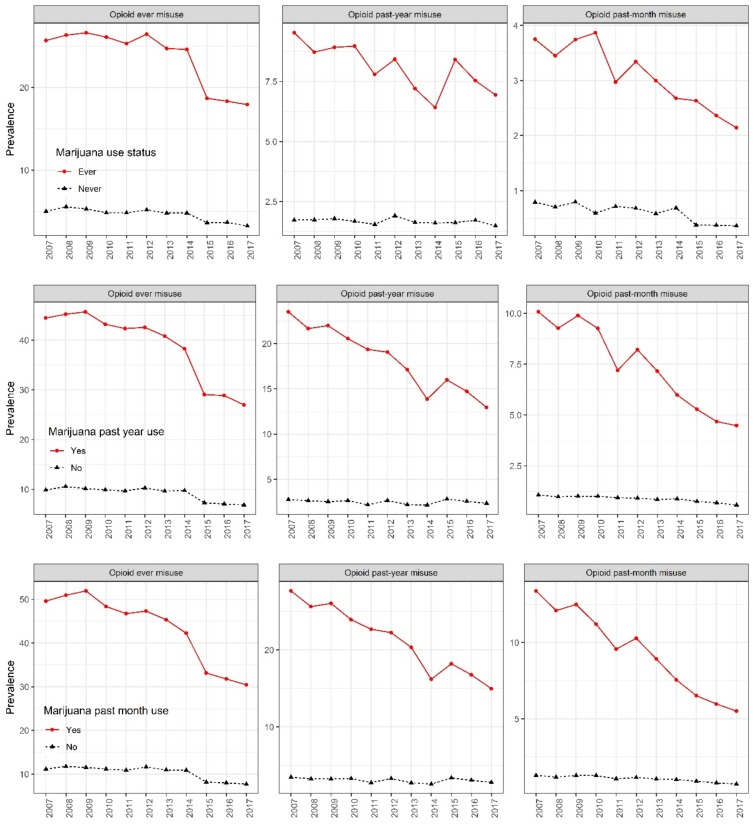
Prevalence of prescription-opioid misuse in the United States stratified by marijuana use status, 2007–2017.

Figure 2 presents the trend of prescription-opioid use stratified by marijuana use status. Across the study period, the percentages of all prescription-opioid misuse categories declined among all subgroups. Among marijuana past-month users, prevalence of ever prescription-opioid misuse (19.1% change), past-year misuse (12.7% change), and past-month misuse (7.9% change) declined across the study period (2007 to 2017) (z = 1324.1, *p* < 0.0001; z = 1454.4, *p* < 0.0001; and z = 2161.8, *p* < 0.0001, respectively). Smaller reductions in prescription-opioid misuse occurred among those who were not past-month marijuana users, with the prevalence of ever prescription-opioid misuse (3.4% change), past-year misuse (0.7% change), and past-month misuse (0.6% change) decreasing across the study period (z = 876.8, *p* < 0.0001; z = 444.2, *p* < 0.0001; and z = 2085.3, *p* < 0.0001, respectively). The trends were similar in other groups characterized by marijuana use status: percentage declines of prescription-opioid misuse were larger among those that indicated marijuana ever-use and marijuana past-year use compared to those in the reference categories.

Table 2 presents results from the multivariable analysis of the association between marijuana use and prescription-opioid misuse. After adjusting for sociodemographic characteristics as well as other substance use, marijuana ever-users had higher odds of opioid ever-misuse (OR: 3.04; 95% CI, 2.68–3.43), past-year misuse (OR: 3.44; 95% CI, 3.00–3.94), and past-month misuse (OR: 4.50; 95% CI, 3.35–6.05). Similar associations were found between past-year marijuana use and opioid ever-misuse (OR: 3.67; 95% CI, 3.23–4.16), misuse in the past year (OR: 2.89; 95% CI, 2.48–3.36), and misuse in the past month (OR: 3.12; 95% CI, 2.07–4.72) and between past-month marijuana use and opioid ever-misuse (OR: 3.04; 95% CI, 2.67–3.46), past-year misuse (OR: 3.24; 95% CI, 2.85–3.68), and past-month misuse (OR: 4.27; 95% CI, 3.34–5.44).

## 4. Discussion

The primary objective of the current study was to examine national trends in prescription-opioid misuse in the last decade among adult marijuana users and non-users in the United States. We found that trends of marijuana use increased throughout the study period, which is consistent with prior research [6,26]. This increase could be partially attributed to the decline in perceived risks of marijuana use and the increased prevalence of legalized marijuana [5,6,9]. In 2017, medical marijuana was legal in 30 states including the District of Colombia (D.C.) and recreational marijuana was legal in nine states, including D.C. [27].

Conversely, we found that misuse of prescription opioids declined during the same period. This finding could, in part, be a result of efforts to reduce opioid prescriptions and use. Prescribing rates peaked in 2010, and decreased by 33% from 2013 to 2018 [28,29,30]. It remains unclear how much of this progress is attributable to enhanced care provider education, improved public awareness, state prescribing policy, and the use of prescription drug monitoring programs [28,31,32,33]. Our finding that marijuana users had greater declines in prescription-opioid misuse compared to non-marijuana users may be due to substitution effects. Patients who would have been prescribed opioids to manage chronic pain may be utilizing medical and recreational marijuana as a pain reliever instead. The implementation of medical marijuana laws has been associated with a 5.88% lower rate of Medicaid-covered opioid prescriptions [34].

Additionally, we found that past-month marijuana users had significantly higher odds of all prescription-opioid use outcomes, although ever-users did not. These odds indicate that more frequent or recent marijuana users are at an increased risk of misusing opioids. Prior studies suggest that a large proportion of marijuana users go on to use other illicit drugs [21]. Distribution of marijuana and other drugs often intersects, providing marijuana users opportunities to obtain other illicit drugs [35,36]. Individuals who are addicted to marijuana are also three times more likely than non-users to be addicted to heroin, and almost 80% of heroin users started with prescription opioids [37,38,39].

We note some limitations. First, the survey relies on self-reported information, which may be subject to inaccurate recall. Second, the cross-sectional nature of the study does not allow us to draw a conclusion about the temporality of association; therefore, we do not claim causation. Despite these limitations, this study fills an important gap in the literature by examining national trends and associations of marijuana use and prescription-opioid misuse in an evolving U.S. state legal environment.

## 5. Conclusions

Throughout the study period, trends of marijuana use increased while prescription-opioid use declined. When stratified by past-month or ever marijuana use, larger declines in prescription-opioid misuse were found among those who indicated marijuana use compared to those who did not. Past-month marijuana users had significantly higher odds of ever, past-month, and past-year prescription opioid use, suggesting a possible link between the two substances. This study provides important insights on trends and associations given the changing social environment of drug use in the United States. These findings underscore the importance of future research to assess whether there is a causal relationship between marijuana use and prescription-opioid misuse.

## Figures and Tables

**Table 1:** Descriptive characteristics of U.S. population aged ≥18: data from the 2016–2017 National Survey on Drug Use and Health (NSDUH).

Variables	Total Sample *n* (%)	Marijuana Never-User *n* (%)	Marijuana Ever-User *n* (%)
Total	85,179 (100.0)	41,628 (52.5)	43,551 (47.5)
Age			
18–25	27,500 (14.0)	13,096 (12.8)	14,404 (15.3)
26–34	17,537 (15.9)	7592 (13.4)	9945 (18.8)
35–49	22,575 (24.8)	10,876 (23.3)	11,699 (26.4)
50+	17,567 (45.3)	10,064 (50.5)	7503 (39.5)
Sex			
Male	39,840 (48.2)	17,859 (43.9)	21,981 (53.1)
Female	45,339 (51.8)	23,769 (56.1)	21,570 (46.9)
Marital status			
Married	35,124 (51.8)	18,887 (55.6)	16,237 (47.5)
Widowed	2488 (5.7)	1790 (8.3)	698 (2.9)
Divorced or separated	9220 (13.8)	3853 (11.7)	5367 (16.2)
Never been married	38,347 (28.7)	17,098 (24.5)	21,249 (33.4)
Race			
Non-Hispanic White	51,839 (64.1)	22,765 (57.3)	29,074 (71.6)
Non-Hispanic Black	10,704 (11.9)	5589 (12.3)	5115 (11.3)
Hispanic	14,240 (15.9)	8598 (19.7)	5642 (11.7)
Other races	8396 (8.1)	4676 (10.6)	3720 (5.4)
Overall health			
Excellent	9761 (21.0)	5381 (22.4)	4380 (19.5)
Very good	16,151 (36.6)	7513 (34.7)	8638 (38.7)
Good	11,800 (28.5)	5481 (28.7)	6319 (28.3)
Fair/poor	4829 (13.9)	2237 (14.2)	2592 (13.5)
Education categories			
Less than high school	10,884 (12.6)	6395 (15.7)	4489 (9.1)
High school graduate	22,575 (24.8)	11,329 (25.9)	11,246 (23.6)
Some college/associates degree	28,723 (31.0)	12,670 (27.5)	16,053 (34.9)
College graduate	22,997 (31.6)	11,234 (30.9)	11,763 (32.4)
Employment status			
Employed full-time	44,410 (49.5)	19,602 (43.4)	24,808 (56.3)
Employed part-time	13,345 (13.0)	6410 (12.2)	6935 (13.9)
Unemployed	5178 (4.4)	2343 (4.0)	2835 (4.9)
Other	22,246 (33.0)	13,273 (40.3)	8973 (24.9)
Total family income			
Less than $20,000	17,309 (16.6)	8723 (17.6)	8586 (15.5)
$20,000–$49,999	26,814 (29.7)	13,476 (31.8)	13,338 (27.4)
$50,000–$74,999	13,247 (15.9)	6386 (15.9)	6861 (16.0)
$75,000 or more	27,809 (37.8)	13,043 (34.8)	14,766 (41.2)
Past year serious psychological distress			
Yes	6620 (11.2)	2172 (7.3)	4448 (15.4)
No	35,934 (88.8)	18,445 (92.7)	17,489 (84.6)
Ever smoked a cigarette			
Yes	51,631 (62.0)	15,532 (41.8)	36,099 (84.4)
No	33,548 (38.0)	26,096 (58.2)	7452 (15.6)
Ever had a drink of alcoholic beverage			
Yes	73,217 (86.0)	30,482 (74.8)	42,735 (98.3)
No	11,948 (14.0)	11,133 (25.2)	815 (1.7)
Ever used crack			
Yes	3022 (23.2)	57 (20.5)	2965 (23.3)
No	10,044 (76.8)	218 (79.5)	9826 (76.7)
Ever used heroin			
Yes	1945 (2.1)	43 (0.1)	1902 (4.3)
No	83,220 (97.9)	41,576 (99.9)	41,644 (95.7)
Ever used methamphetamine			
Yes	4991 (6.0)	150 (0.4)	4841 (12.2)
No	80,136 (94.0)	41,443 (99.6)	38,693 (87.8)
Prescription-opioid misuse ever-use			
Yes	10,406 (10.5)	1623 (3.6)	8783 (18.2)
No	74,773 (89.5)	40,005 (96.4)	34,768 (81.8)
Prescription-opioid past-year misuse			
Yes	4556 (4.2)	772 (1.6)	3784 (7.1)
No	80,623 (95.8)	40,856 (98.4)	39,767 (92.9)
Prescription-opioid past-month misuse			
Yes	1314 (1.2)	157 (0.4)	1157 (2.2)
No	83,865 (98.8)	41,471 (99.6)	42,394 (97.8)

All descriptive statistics were presented in unweighted counts and weighted column percentage except for the Total column in which we used the row percentage. Rao-Scott Chi-Square tests were used for comparing characteristics and all test results were significant (*p* < 0.0001). Numbers may not sum to totals because of missing data.

**Table 2:** Association between prescription-opioid misuse and marijuana past-month use status.

Marijuana Use	Prescription-Opioid Ever Misuse OR (95% CI)	Prescription-Opioid Past-Year Misuse OR (95% CI)	Prescription-Opioid Past-Month Misuse OR (95% CI)
Ever use			
Yes	**3.04 (2.68–3.43)**	**3.44 (3.00–3.94)**	**4.50 (3.35–6.05)**
No	Ref	ref	Ref
Past-year use			
Yes	**3.67 (3.23–4.16)**	**2.89 (2.48–3.36)**	**3.12 (2.07–4.72)**
No	Ref	ref	Ref
Past-month use			
Yes	**3.04 (2.67–3.46)**	**3.24 (2.85–3.68)**	**4.27 (3.34–5.44)**
No	Ref	ref	Ref

Ref = reference category and significant odds ratios are presented in bold. Multivariable logistic regression was used in examining the association between prescription-opioid and marijuana use adjusting for age, sex, race, overall health, education, employment status, family income, survey year, past-year serious psychological distress, and substance use, including cigarettes and alcohol.

## References

[1] U.S. Department of Health and Human Services HHS Acting Secretary Declares Public Health Emergency to Address National Opioid Crisis. https://www.hhs.gov/about/news/2017/10/26/hhs-acting-secretary-declares-public-health-emergency-address-national-opioid-crisis.html.

[2] National Institute on Drug Abuse Prescription Opioid Use Is a Risk Factor for Heroin Use. https://www.drugabuse.gov/publications/research-reports/relationship-between-prescription-drug-heroin-abuse/prescription-opioid-use-risk-factor-heroin-use.

[3] Substance Abuse and Mental Health Services Administration (2018). Key Substance Use and Mental Health Indicators in the United States: Results from the 2017 National Survey on Drug Use and Health (HHS Publication No. SMA 18-5068, NSDUH Series H-53).

[4] Cragg A., Hau J.P., Woo S.A., Kitchen S.A., Liu C., Doyle-Waters M.M., Hohl C.M. (2019). Risk Factors for Misuse of Prescribed Opioids: A Systematic Review and Meta-Analysis. Ann. Emerg. Med..

[5] Tzilos G.K., Reddy M.K., Caviness C.M., Anderson B.J., Stein M.D. (2014). Getting higher: Co-occurring drug use among marijuana using emerging adults. J. Addict. Dis..

[6] Carliner H., Mauro P.M., Brown Q.L., Shmulewitz D., Rahim-Juwel R., Sarvet A.L., Wall M.M., Martins S.S., Carliner G., Hasin D.S. (2017). The widening gender gap in marijuana use prevalence in the U.S. during a period of economic change, 2002–2014. Drug Alcohol Depend..

[7] National Conference of State Legislatures State Medical Marijuana Laws. http://www.ncsl.org/research/health/state-medical-marijuana-laws.aspx.

[8] Abuse N.I. On D. Marijuana. https://www.drugabuse.gov/publications/drugfacts/marijuana.

[9] Patton G.C., Coffey C., Carlin J.B., Degenhardt L., Lynskey M., Hall W. (2002). Cannabis use and mental health in young people: Cohort study. BMJ.

[10] Di Forti M., Quattrone D., Freeman T.P., Tripoli G., Gayer-Anderson C., Quigley H., Rodrigues V., Jongsma H.E., Ferraro L., La Cascia C. (2019). The contribution of cannabis use to variation in the incidence of psychotic disorder across Europe (EU-GEI): A multicentre case-control study. Lancet Psychiatry.

[11] Bachhuber M.A., Saloner B., Cunningham C.O., Barry C.L. (2014). Medical Cannabis Laws and Opioid Analgesic Overdose Mortality in the United States, 1999–2010. JAMA Intern. Med..

[12] Shover C.L., Davis C.S., Gordon S.C., Humphreys K. (2019). Association between medical cannabis laws and opioid overdose mortality has reversed over time. Proc. Natl. Acad. Sci. USA.

[13] Segura L.E., Mauro C.M., Levy N.S., Khauli N., Philbin M.M., Mauro P.M., Martins S.S. (2019). Association of US Medical Marijuana Laws with Nonmedical Prescription Opioid Use and Prescription Opioid Use Disorder. JAMA Netw. Open.

[14] Bradford A.C., Bradford W.D. (2017). Medical Marijuana Laws May Be Associated with a Decline in the Number of Prescriptions for Medicaid Enrollees. Health Aff. Proj. Hope.

[15] Piper B.J., DeKeuster R.M., Beals M.L., Cobb C.M., Burchman C.A., Perkinson L., Lynn S.T., Nichols S.D., Abess A.T. (2017). Substitution of medical cannabis for pharmaceutical agents for pain, anxiety, and sleep. J. Psychopharmacol. Oxf. Engl..

[16] Raby W.N., Carpenter K.M., Rothenberg J., Brooks A.C., Jiang H., Sullivan M., Bisaga A., Comer S., Nunes E.V. (2009). Intermittent marijuana use is associated with improved retention in naltrexone treatment for opiate-dependence. Am. J. Addict..

[17] Scavone J.L., Sterling R.C., Weinstein S.P., Van Bockstaele E.J. (2013). Impact of cannabis use during stabilization on methadone maintenance treatment. Am. J. Addict..

[18] Caputi T., Humphreys K. (2018). Medical Marijuana Users are More Likely to Use Prescription Drugs Medically and Nonmedically. J. Addict. Med..

[19] Campbell G., Hall W.D., Peacock A., Lintzeris N., Bruno R., Larance B., Nielsen S., Cohen M., Chan G., Mattick R.P. (2018). Effect of cannabis use in people with chronic non-cancer pain prescribed opioids: Findings from a 4-year prospective cohort study. Lancet Public Health.

[20] Agrawal A., Neale M.C., Prescott C.A., Kendler K.S. (2004). A twin study of early cannabis use and subsequent use and abuse/dependence of other illicit drugs. Psychol. Med..

[21] Secades-Villa R., Garcia-Rodríguez O., Jin C.J., Wang S., Blanco C. (2015). Probability and predictors of the cannabis gateway effect: A national study. Int. J. Drug Policy.

[22] Center for Behavioral Health Statistics and Quality (2015). Behavioral Health Trends in the United States: Results from the 2014 National Survey on Drug Use and Health.

[23] Center for Behavioral Health Statistics and Quality Substance Abuse and Mental Health Services Administration 2017 National Survey on Drug Use and Health: Methodological Resource Book. https://www.samhsa.gov/data/report/nsduh-2017-methodological-resource-book-mrb..

[24] American Psychiatric Association Diagnostic and Statistical Manual of Mental Disorders, 4th ed. https://dsm.psychiatryonline.org/doi/abs/10.1176/appi.books.9780890420249.dsm-iv-tr..

[25] Kessler R.C., Barker P.R., Colpe L.J., Epstein J.F., Gfroerer J.C., Hiripi E., Howes M.J., Normand S.-L.T., Manderscheid R.W., Walters E.E. (2003). Screening for serious mental illness in the general population. Arch. Gen. Psychiatry.

[26] Hasin D.S., Saha T.D., Kerridge B.T., Goldstein R.B., Chou S.P., Zhang H., Jung J., Pickering R.P., Ruan W.J., Smith S.M. (2015). Prevalence of Marijuana Use Disorders in the United States Between 2001-2002 and 2012-2013. JAMA Psychiatry.

[27] ProCon 33 Legal Medical Marijuana States and DC—Medical Marijuana—ProCon.org. https://medicalmarijuana.procon.org/view.resource.php?resourceID=000881.

[28] American Medical Association (2019). Physicians’ progress toward ending the nation’s opioid epidemic. Opioid Task Force 2019 Progress Report.

[29] Kuehn B. (2019). Declining Opioid Prescriptions. JAMA.

[30] Guy G.P. (2017). Vital Signs: Changes in Opioid Prescribing in the United States, 2006–2015. MMWR Morb. Mortal. Wkly. Rep..

[31] Murthy V.H. (2016). Ending the Opioid Epidemic—A Call to Action. N. Engl. J. Med..

[32] Penm J., MacKinnon N.J., Boone J.M., Ciaccia A., McNamee C., Winstanley E.L. (2017). Strategies and policies to address the opioid epidemic: A case study of Ohio. J. Am. Pharm. Assoc..

[33] Dowell D., Haegerich T.M., Chou R. (2016). CDC Guideline for Prescribing Opioids for Chronic Pain—United States, 2016. MMWR Recomm. Rep..

[34] Wen H., Hockenberry J.M. (2018). Association of Medical and Adult-Use Marijuana Laws with Opioid Prescribing for Medicaid Enrollees. JAMA Intern. Med..

[35] Wagner F.A., Anthony J.C. (2002). Into the world of illegal drug use: Exposure opportunity and other mechanisms linking the use of alcohol, tobacco, marijuana, and cocaine. Am. J. Epidemiol..

[36] Centers for Disease Control and Prevention Today’s Heroin Epidemic Infographics 2015. https://www.cdc.gov/vitalsigns/heroin/infographic.html.

[37] Monico L.B., Mitchell S.G. (2018). Patient perspectives of transitioning from prescription opioids to heroin and the role of route of administration. Subst. Abuse Treat. Prev. Policy.

[38] Compton W.M., Jones C.M., Baldwin G.T. (2016). Relationship between Nonmedical Prescription-Opioid Use and Heroin Use. N. Engl. J. Med..

[39] Muhuri P.K., Gfroerer J.C., Davies M.C. CBHSQ Data Review: Associations of Nonmedical Pain Reliever Use and Initiation of Heroin Use in the United States. https://www.samhsa.gov/data/sites/default/files/DR006/DR006/nonmedical-pain-reliever-use-2013.htm.

